# Serotype Distribution and Pathotypic Characteristics of *Streptococcus suis* Isolates from Slaughtered Pigs in a High-Density Pig Farming Area in Thailand

**DOI:** 10.1155/2024/3186518

**Published:** 2024-07-10

**Authors:** Kamonwan Lunha, Wiyada Chumpol, Surasak Jiemsup, Suganya Yongkiettrakul, Jinquan Li, Anusak Kerdsin, Daisuke Takamatsu, Nattakan Meekhanon

**Affiliations:** ^1^ National Center for Genetic Engineering and Biotechnology National Science and Technology Development Agency, Pathum Thani 12120, Thailand; ^2^ State Key Laboratory of Agricultural Microbiology Key Laboratory of Environment Correlative Dietology College of Food Science and Technology Shenzhen Institute of Nutrition and Health Huazhong Agricultural University, Wuhan 430070, China; ^3^ Faculty of Public Health Kasetsart University Chalermphrakiat Sakon Nakhon Province Campus, Sakon Nakhon 47000, Thailand; ^4^ Bacterial and Parasitic Diseases Research Division National Institute of Animal Health National Agriculture and Food Research Organization, Tsukuba, Ibaraki 305-0856, Japan; ^5^ The United Graduate School of Veterinary Sciences Gifu University, Gifu 501-1193, Japan; ^6^ Joint Graduate School of Veterinary Sciences Gifu University, Gifu 501-1193, Japan; ^7^ Department of Veterinary Nursing Faculty of Veterinary Technology Kasetsart University, Bangkok 10900, Thailand

## Abstract

*Streptococcus suis* is a significant bacterial pathogen in the swine industry and represents a zoonotic threat to human health. This study aimed to investigate the prevalence, serotype distribution, and pathotypic characteristics of *S. suis* isolates obtained from nasopharyngeal swabs of slaughtered pigs in the high-density swine farming region of Thailand. Among 322 swab samples, 194 samples (60.25%) were found to harbor *S. suis*. The most prevalent serotype was serotype 8 (7.98%), followed by 19 (7.56%), 29 (6.72%), 3 (5.88%), and 2 (5.04%), with 39.92% of isolates classified as non-typeable. Molecular characterization revealed the presence of various clonal complexes (CCs), with CC221/234 being the most prevalent (19.15%). Human-associated clades (HAC) were identified in 29.79% of isolates, including serotypes 2 (two isolates), 1/2 (two isolates), and 4 (four isolates) in CC233/379. Additionally, several isolates exhibited a high potential for zoonotic transmission, particularly within the CC233/379 clade, which emerged exclusively in Thailand. Molecular pathotyping uncovered challenges in differentiating pathogenic and commensal *S. suis* strains in healthy pigs. Despite this, surveillance and monitoring of *S. suis* populations are essential to track dynamics and mitigate the risk of human infections.

## 1. Introduction

*Streptococcus suis* is an important bacterial pathogen that poses a considerable threat to the swine industry worldwide. It can cause various diseases in pigs, including meningitis, septicemia, endocarditis, pneumonia, and arthritis [1]. This pathogen can be transmitted to humans through direct contact with infected pigs or contaminated pork and consumption of undercooked or raw pork dishes. The most frequent clinical manifestation of human infection is septicemia, followed by meningitis and endocarditis, and hearing loss is a known complication associated with *S. suis* meningitis [2]. Pathogenic strains of *S. suis* can cause disease and are typically associated with clinical manifestations, on the other hand, commensal *S. suis* strains can be found in the respiratory and gastrointestinal tracts of healthy pigs without causing disease [3]. *S. suis* can also act as an opportunistic pathogen which can cause infections when the predisposing factors are present.

Serotyping and multilocus sequence typing (MLST) are commonly used to identify the pathogenic *S. suis* strains. Serotyping of *S. suis* is based on the capsular polysaccharide antigens, which exhibit significant diversity. Among 29 serotypes that have been described to date, serotype 2 is the most frequently associated with *S. suis* infections in both pigs and humans. Serotypes 4, 5, 9, 14, 16, 21, 24, and 31 have also been reported in human infections, while serotypes 1/2, 3, 7, 9, and 14 are commonly associated with diseased pigs [1, 4, 5].

MLST is a molecular typing method used to determine the genetic relatedness and population structure of *S. suis* strains. The sequence types (STs) obtained through MLST can be utilized to determine the clonal complexes (CCs) which are groups of genetically closely related strains. The MLST analysis of *S. suis* has revealed the presence of different CCs, with CC1, CC16, CC20, CC25, CC28, CC104, CC233/379, and CC221/234 being among the most important causes of infections [5, 6]. The prevalence of specific serotypes and CCs can vary significantly across different geographical regions and may also undergo changes over time. As a result, it is crucial to conduct regular surveillance and monitoring to track any shifts in the *S. suis* population.

The PCR-based pathotyping is a recently developed molecular technique used to distinguish isolates into disease-associated and non-disease-associated groups [7]. When coupled with human-associated clade (HAC) markers [8], this technique has demonstrated potential as a valuable and cost-effective tool for identifying high-risk *S. suis* strains. Additionally, the identification of HAC markers further enhances the capability to predict zoonotic potential strains.

In Thailand, Nakhon Pathom and Ratchaburi provinces serve as major hubs for the pig farming industry and play a crucial role in pork production. Moreover, their strategic proximity to Bangkok, the capital city of Thailand, facilitates the efficient transportation and distribution of pork, catering to both local and national demands. Since *S. suis* can be transmitted through direct occupational contact and pork consumption, the study of *S. suis* population within these areas becomes a matter of great concern. Therefore, in this study, we aimed to investigate the serotype distribution and pathotypic characteristics of *S. suis* isolates obtained from slaughtered pigs in this high-density area of pig production. Understanding and monitoring the prevalent serotypes and dynamics of potentially hazardous *S. suis* strains in these regions can help in implementing targeted measures to mitigate potential health risks and safeguard public health.

## 2. Materials and Methods

### 2.1. Sample Collection

Nasopharyngeal swab samples were collected from 322 slaughtered pigs at three slaughterhouses located in Nakhon Pathom province during two distinct periods: April to May and October to November 2022. These pigs originated from various farms situated in Nakhon Pathom and Ratchaburi provinces. To ensure proper collection, a swab was inserted into the nasopharyngeal area on both sides of each pig and then preserved in commercial Stuart transport medium (Yangzhou Chuangxin Device Factory, Jiangsu, China). Immediately after collection, the samples were transported under cool conditions to the microbiology laboratory at the Faculty of Veterinary Technology, Kasetsart University.

### 2.2. *S. suis* Isolation and Identification

The collected swab samples were inoculated on Todd–Hewitt agar (THA) (HiMedia, Mumbai, India) supplemented with Streptococcal Selective Supplement (Oxoid, Hampshire, UK) and then placed in an incubator at 37°C with 5% CO_2_ for a 24-hr period. Subsequently, a maximum of six streptococcus-like colonies observed on each plate were selected and subcultured onto fresh THA plates. After the incubation, the bacterial isolates with small (about 1 mm in diameter), gray-white, opaque, and round colonies were classified into the presumptive *S. suis* by Gram-positive staining and catalase-negative results. The genomic DNA of each selective isolate was then extracted using InstaGene Matrix (Bio-Rad, CA, USA), following the instructions provided by the manufacturer.

Species-specific PCR targeting the recombination/repair protein (*recN*) gene was employed to identify *S. suis* isolates, following the previously described method [9]. The primers used in this PCR were as follows: the forward primer sequence was 5′-TTATCTGTCTTGAAACAGATTGGG-3′, and the reverse primer sequence was 5′-TCTTTCTCTAAGTTCTTAAGCTGAAC-3′. The PCR reactions were performed using *Taq* DNA polymerase with a standard *Taq* buffer (New England BioLabs, MA, USA), following the manufacturer's instructions. The PCR program included a predenaturation step at 95°C for 5 min, followed by 35 cycles of denaturation at 95°C for 30 s, annealing at 54°C for 1 min, and extension at 72°C for 1 min. A final extension step was performed at 72°C for 7 min. After the PCR amplification, the PCR products were subjected to electrophoresis on 1.5% agarose gels, and the gels were stained with SYBR™ Safe (Invitrogen, CA, USA) and visualized under ultraviolet light. The expected size of the PCR product was 731 base pairs, providing a specific indication of the presence of the target *S. suis* isolates.

### 2.3. Capsular Typing and Pathotypic Characterization

The serotypes of *S. suis* isolates were determined using a multiplex PCR method, previously described in the study conducted by Kerdsin et al. [10]. Briefly, the PCR reactions were divided into four independent sets, each targeting specific serotypes (Table S1). The first set of PCR reactions included primers for serotypes 1/2, 1, 2, 3, 7, 9, 11, 14, and 16. The second set of PCR reactions targeted serotypes 4, 5, 8, 12, 18, 19, 24, and 25. The third set of PCR reactions focused on serotypes 6, 10, 13, 15, 17, 23, and 31. Lastly, the fourth set of PCR reactions was designed for serotypes 21, 27, 28, 29, and 30. The PCR reactions were performed using the following conditions: an initial denaturation at 95°C for 3 min, followed by 30 cycles of 95°C for 20 s, 62°C for 1 min and 30 s, and 72°C for 1 min and 30 s. A final extension step was conducted at 72°C for 5 min. The differentiation of serotype 2 and 1/2 was confirmed through the examination of a single nucleotide substitution of a glycosyl transferase gene (*cpsK*) gene, which serotype 2 possesses G in contrast to serotype 1/2 with C or T at position 483 [11].

*S. suis* isolates representing each serotype were randomly selected for further analysis of three pathotypic characters, consisting of clonal complex (CC) identification, the presence of disease-associated and human-associated markers. Unfortunately, due to an unexpected loss of bacterial viability within the storage, isolates categorized under serotypes 12, 15, 21, and 27 could not undergo further analysis.

The multiplex PCR method was employed to identify the major CCs associated with human infections, including CC1, CC25, CC28, CC104, CC221/234, and CC233/379. The reaction was conducted in accordance with the methods described in the previous study [12], utilizing five pairs of primers designed to target the *hp1*, *mrp*, *col*, *pep*, and *srtBCD* genes. The primer sequences used in this study are listed in Table S2.

PCR-based pathotyping used to distinguish disease-associated and non-disease-associated strains was performed as previously described [7, 13]. The primer sequences are listed in Table S3. The characterization of HAC was conducted by PCR method using HAC17279-membrane protein gene marker as described in a recent study [8] with distinct HAC-specific PCR primers (Table S4).

Correlation between the serotype and CC to the HAC statuses was investigated by performing Pearson's correlation analysis with the STATA statistical package v14.0 (Stata Corporation, College Station, TX, USA) under a significant *p*-value of <0.05.

### 2.4. Comparison of Serotype Distribution

A descriptive analysis was conducted to compare the serotype distribution of *S. suis* isolates identified in this current study with that of a prior study [14] involving pigs from a comparable geographical area. The samples in this current study were collected in 2022, while those for comparison were obtained from a study conducted between 2014 and 2015. Therefore, this study examined the evolving serotype distribution over a period of at least 7 years. The Pearson's chi-square test was used to compare the prevalence of various serotypes in different study periods. A *p*-value of <0.05 was considered significant.

## 3. Results

### 3.1. Prevalence of *S. suis* in Nasopharyngeal Swabs of Slaughtered Pigs

A total of 238 *S. suis* isolates were retrieved from nasopharyngeal swabs obtained from slaughtered pigs. Out of the 322 nasopharyngeal swab samples, 194 were determined to harbor *S. suis*, representing a prevalence of 60.25%. This rate is notably higher than the carriage rate reported in a previous study [14], which was used for data comparison and reported a prevalence rate of 37%.

Out of the 194 positive samples, a single isolate was obtained from each sample in 155 cases (79.89%). Additionally, isolates with two and three different serotypes were retrieved from 34 samples (17.53%) and five samples (2.58%), respectively.

### 3.2. Comparison of Serotype Distribution of *S. suis* Isolated between 2014–2015, and 2022

Based on the PCR capsular typing, the most prevalent serotype identified in 2022 was serotype 8 (7.98%, 19/238), followed by 19 (7.56%, 18/238), 29 (6.72%, 16/238), 3 (5.88%, 14/238), and 2 (5.04%, 12/238), respectively. *S. suis* isolates, initially characterized as serotype 1/2 or 2 through the PCR method (14 isolates), were distinctly differentiated by aligning nucleotide sequences of the *cpsK* gene (Figure S1). Among them, isolates YM107 and YM128 were identified as serotype 1/2, while the remaining isolates were classified as serotype 2. Among the total of 238 isolates, 95 isolates were identified as non-typeable (NT) serotype, representing 39.92% of isolates. This finding closely aligns with a previous study in 2014–2015 [14], which reported 43.06% NT isolates. Statistically significant differences were observed in the serotype distribution of *S. suis* isolates between 2022 and those from 2014 to 2015 for the serotypes 4, 5, 15, 16, 19, and 29 (*p*-value < 0.05, Table 1).

As shown in Table 1, the prevalence rates of serotypes 3, 8, 21, 28, and 31 observed in 2022 closely resembled those reported in 2014–2015. Interestingly, the serotype 16 observed in 2022 was notably less prevalence than that found in 2014–2015 (*p*-value < 0.001). The distribution of serotypes isolated in 2022 exhibited greater diversity compared to those from 2014 to 2015. Specifically, we identified serotypes 1/2, 4, 5, 7, 12, 23, 27, and 30 in *S. suis* isolates from 2022, whereas these serotypes were not present in isolates from 2014 to 2015. Conversely, serotypes 10 and 17 were exclusively found in isolates from 2014 to 2015. Serotypes 1, 6, 13, 14, and 25 were not detected in either 2014–2015 or 2022.

### 3.3. Pathotypic Characteristics of *S. suis* from Slaughtered Pigs

Out of 238 *S. suis* isolates, 141 representative isolates displaying various serotypes in 2022 were randomly selected for pathotypic characterization, which included determining CC and assessing the presence of disease-associated and human-associated markers. The results demonstrated a high predominance of CC221/234 (19.15%, 27/141), followed by CC1 (9.93%, 14/141), CC233/379 (5.67%, 8/141), CC28 (3.55%, 5/141), and CC25 (1.42%, 2/141). Among the notable CC221/234 isolates, serotypes 8 and 19 were equally distributed as the most prevalent (29.63%, 8/27 each), followed by serotype 29 (22.22%, 6/27) and NT (18.52%, 5/27). In CC1, serotypes 2 and 9 (28.57%, 4/14 each) and NT (42.86%, 6/14) were identified, while the CC233/379 included serotypes 1/2 and 2 (25%, 2/8 each) and 4 (50%, 4/8). The CC25 and CC28 comprised serotype 3 (two isolates) and serotype 8 (five isolates), respectively. The remaining 85 isolates, including NT serotype (52.94%, 45/85), serotype 19 (5.88%, 5/85), serotypes 5, 7, 9, 28, and 31 (4.71%, 4/85 each), serotypes 3 and 4 (3.53%, 3/85 each), serotype 18 (2.35%, 2/85), and serotype 8, 11, 16, 23, 24, 29, and 30 (1.18%, 1/85 each), were not categorized into any specific CCs using the PCR method employed in this study (Table 2).

The PCR-based pathotyping demonstrated that 117 isolates with various serotypes and CCs were classified as a disease-associated group (Table 3). However, 21 isolates, which included serotypes 4, 8, 28, and NT isolates, displayed specific PCR bands indicative of both disease-associated and non-disease-associated characteristics, leading to their characterization as the undetermined group. Notably, only three isolates, consisting of one serotype 11 and two NT isolates, were non-disease-associated. There were no significant correlations between bacterial serotypes, CCs, and PCR pathotype results of *S. suis*.

Regarding the classification of HACs, 42 out of 141 representative isolates (29.79%) were categorized as human-associated (HA) isolates, while 99 isolates (70.21%) were identified as non-HA isolates. Notably, all CC233/379 isolates (eight isolates, *r* = 0.377), which included serotypes 1/2 and 2 (2 isolates each, *r* = 0.184), as well as serotype 4 (4 isolates, *r* = 0.262), along with all CC25 and CC28 isolates (*r* = 0.184 and 0.294, respectively) identified in this study, were categorized as HA isolates. In addition, all CC1 isolates of which consisted of serotypes 2, 9, and NT were classified as non-HA isolates (data not shown). While CC221/234 was the most prevalent isolates identified in 2022, a portion of them (25.93%, 7/27), including six isolates of serotype 19 (*r* = 0.377) and one isolate of NT, exhibited HA characteristics. Out of 82 unknown CCs, 13 HA isolates, including serotype 19 (four isolates, *r* = 0.294), serotypes 4, 5, 7, 9, 23, and 28 (one isolate each), and NT (three isolates) were identified. The results of serotypes, CCs, and HAC for the 141 representative isolates were presented in Tables 4 and S5.

## 4. Discussion

*S. suis* is a major swine pathogen with the greatest impact on pig production worldwide. It has considerable zoonotic potential for humans, especially in East and South-East Asian countries including China, Vietnam, and Thailand [15]. In this study, using nasopharyngeal swab samples, a significantly high prevalence rate of *S. suis* (60.25%) was observed in the slaughtered pig population in Nakhon Pathom and Ratchaburi provinces, central Thailand compared to a previous study (37%) [14]. It is noteworthy that the sample collection in this study was restricted to nasopharyngeal swabs from slaughtered pigs which may be considered as a partial sterile site. In contrast, the previous study included samples from both live and slaughtered pigs, incorporating nonsterile sites like saliva, nasal, and oral swabs. The broader sampling approach may have introduced more contamination with various other bacteria, posing challenges in isolating *S. suis* and potentially resulting in the lower prevalence rate of *S. suis* observed in the previous study. In a previous study by Kerdsin et al. [4], *S. suis* was reported in tonsils collected from slaughtered pigs in Phayao province, northern Thailand, at a rate of 35.2%. Additionally, significant variations in the prevalence of *S. suis* in pigs become evident when considering data from different countries. For example, in Vietnam, there was a reported prevalence rate of 2.3% in swine oral swab samples [16], while in India and Korea, tonsil samples showed prevalence rates of 32.7% [17] and 13.8% [18], respectively. Thus, the variations in sample sources, identification method, and geographical differences may contribute to differences in prevalence rate of *S. suis*.

When specifically examining the carriage rate of serotype 2, a significant serotype of *S. suis* responsible for human infection, the difference in prevalence of *S. suis* serotype 2 between nasal swabs and tonsils becomes evident. In tonsil samples collected from Phayao province (northern Thailand), previous studies reported serotype 2 at rates of 6.7% [4] and 6% [19]. However, this study found a lower serotype 2 carriage rate of 3.7% (12/322) in nasopharyngeal swab samples from central regions of Thailand, suggesting a distinct geographic distribution pattern of serotype 2 among healthy pig populations. The higher prevalence of serotype 2 in tonsil samples, compared to nasal swabs, may be attributed to tonsils acting as specific sites for *S. suis* infection. Nevertheless, the presence of serotype 2 in nasal swabs from healthy pig populations implies an elevated risk of human infection.

According to the results of capsular typing in the present study, 22 serotypes were identified, demonstrating highly variable prevalence rates (0.4%−7.9%) and highlighting the high diversity of *S. suis* in healthy slaughter pigs in Thailand. Notably, a substantial portion of the isolates (39.9%) were identified as NT, aligning with findings from previous studies in central (43%) and northern (37.2%) regions of Thailand [14, 19]. These evidences suggested potential challenges in traditional capsular typing methods or the emergence of novel capsular types. Therefore, further whole-genome sequencing analysis is required to understand the genetic characteristics of *cps* loci in NT *S. suis* strains.

The prevalence of serotypes 3, 8, 21, 28, and 31 observed in the current study closely resembled that reported in the earlier study [14], suggesting a notable stability in the existence of these serotypes over time. This study significantly revealed a higher prevalence of serotype 16 in 2014–2015 compared to that in 2022. In addition, several serotypes (1/2, 4, 5, 7, 12, 23, 27, and 30) that were absent in the isolates from 2014 to 2015 were identified in 2022. Conversely, serotypes 10 and 17 were exclusively found in isolates from 2014 to 2015. These findings suggested a potential shift in the prevalence of different *S. suis* serotypes in the swine population, as well as changes in farm setting conditions introducing alterations in the microbial landscape and health dynamics of pigs. The changing serotype distribution of *S. suis* isolates over time is of significant epidemiological interest, reflecting shifts in bacterial genetic diversity, host populations, and/or environmental factors.

The presence of multiple *S. suis* serotypes in the same pig over the same period of time suggested the ability of coexistence or coinfection of *S. suis* strains with distinct genetic diversity. This scenario could have implications for the overall dynamics of bacterial community within the host, potentially influencing the severity of the infection. Further investigation would be warranted to understand the implications of such coexistence and its potential impact on animal health.

The stable persistence of *S. suis* in the tonsil niche has been reported [20]. However, the genetic diversity of *S. suis* isolates within the nasal cavity has the potential to influence strain prevalence and disease progression. Furthermore, high genetic diversity provides *S. suis* with the potential to evolve rapidly, leading to the emergence of new strains with varied characteristics. These changes may include alterations in virulence, antibiotic resistance, and fitness, enabling the bacteria to thrive and persist in their environment. Hence, understanding this genetic diversity is of utmost importance, as it can play a pivotal role in determining the severity of disease outbreaks within swine populations and can also influence the effectiveness of treatment protocols, including antibiotic therapies.

Differentiating between pathogenic and commensal *S. suis* strains requires a comprehensive assessment relating various factors, which include evaluating the presence or absence of specific virulence factors, analyzing serotype distribution patterns, conducting genetic characterization, and evaluating actual phenotypes using animal models and *in vitro* analytical methods. In this study, PCR-based methods were employed to assess the presence of crucial CCs associated with human infections in Thailand and to classify *S. suis* isolates as either human-associated (HA) or non-human-associated (non-HA) isolates. Among the 56 out of 141 representative isolates for which the CCs were identifiable, CC221/234 was predominantly detected across various serotypes, including serotypes 8, 19, 29, and NT isolates. It is noteworthy that, although CC221/234-serotype 19 has not been previously reported in human infections, eight isolates of CC221/234-serotype 19 found in this study were classified as HA isolates. Furthermore, two isolates of CC25-serotype 3 and five isolates of CC28-serotype 8 were classified as HA isolates. As reported in a previous study [21], the serotype-switch mutant to serotype 8 demonstrated increased virulence. Strains belonging to CC221/234-serotype 8 and CC28-serotype 8 may present a significant risk and require close monitoring.

The recent review on *S. suis* isolated from human infections in Thailand has demonstrated that *S. suis* isolates within CC221/234 were predominantly characterized as serotypes 5, 24, and 31, while CC25 and CC28 have been associated with serotype 2 [6]. Therefore, it is crucial to closely monitor the potential pathogenicity and zoonotic transmission of serotypes 3, 8, and 19. Additionally, considering a previous study of capsule switching in serotype 2 [22], there is a possibility that a similar phenomenon has occurred within the serotypes of CC25 and CC28.

The present study identified *S. suis* isolates with a high potential for zoonotic transmission, including CC25, CC28, and CC233/379, as they were categorized into HAC. The CC233/379 has emerged exclusively in Thailand over the last two decades. Recently, the strains responsible for a recent human outbreak of *S. suis* in Nakhon Ratchasima province, Thailand, were identified as belonging to the CC233/379 clade [23]. This highlights the evolving nature of *S. suis* strains and their potential to pose a threat to human health.

It is important to note that only three isolates clearly showed negative results for pathotype identification with the specified marker genes (Table 3) and considering that the *S. suis* strains isolated from clinically healthy pigs, the numbers of isolates with pathotypes seemed to be unexpectedly high. This is consistent with a previous study that has reported the limitation of using the multiplex PCR-based pathotyping method to differentiate *S. suis* isolates from healthy pigs [13]. The molecular pathotyping method should be used with caution regarding a high genetic diversity of *S. suis* strains from different sample sources and/or geographical locations.

A high prevalence of *S. suis* observed in the nasal passages of slaughtered pigs in a densely populated swine area in Thailand revealed dynamic changes in the serotype distribution of the *S. suis* population when compared to data from a previous study conducted in the same area. Furthermore, strains exhibiting a high potential for zoonotic transmission among the isolates have been investigated. The elevated prevalence discovered in our study indicates the widespread of *S. suis* within the nasal passages of slaughtered pigs, thus presenting significant concerns for the swine industry and food safety.

## 5. Conclusion

This study reported a high prevalence of *S. suis* in the nasal passages of slaughtered pigs in a swine-dense area of Thailand. The dynamic changes in the serotype distribution of *S. suis* over time were observed. Additionally, the CCs and HAC classification demonstrated that the *S. suis* CC233/379 isolates from healthy slaughtered pig possessed a high potential for zoonotic transmission. The findings highlight the necessity for targeted interventions and rigorous biosecurity measures to mitigate the risk of human infections, protecting both swine industry workers and consumers of pork products.

## Figures and Tables

**Table 1:** The distribution of serotypes isolated in 2022 compared to those in 2014–2015, *n* (%).

Serotype	Year		*p* -Value^a^
	2014–2015 (*n* = 137)	2022 (*n* = 238)	
1/2	—	2 (0.84)	0.282
2	4 (2.92)	12 (5.04)	0.327
3	7 (5.11)	14 (5.88)	0.754
4	—	8 (3.36)	**0.030**
5	—	7 (2.94)	**0.043**
7	—	5 (2.1)	0.088
8	10 (7.3)	19 (7.98)	0.811
9	8 (5.84)	8 (3.36)	0.253
10	2 (1.46)	—	0.062
11	5 (3.65)	3 (1.26)	0.123
12	—	1 (0.42)	0.447
15	5 (3.65)	1 (0.42)	**0.016**
16	16 (11.68)	3 (1.26)	**0.000**
17	2 (1.46)	—	0.062
18	5 (3.65)	3 (1.26)	0.123
19	3 (2.19)	18 (7.56)	**0.029**
21	2 (1.46)	4 (1.68)	0.87
23	—	1 (0.42)	0.447
24	1 (0.73)	3 (1.26)	0.63
27	—	2 (0.84)	0.282
28	4 (2.92)	7 (2.94)	0.991
29	2 (1.46)	16 (6.72)	**0.022**
30	—	2 (0.84)	0.282
31	2 (1.46)	4 (1.68)	0.87
Non-typeable	59 (43.06)	95 (39.92)	0.551

^a^Numbers in boldface represent statistically significant different (*p*-value < 0.05).

**Table 2:** Correlation between serotypes and clonal complexes in representative 141 *S. suis* isolates in 2022, *n* (%).

Serotype	Clonal complex (CC)						Total (*n* = 141)
	CC1 (*n* = 14)	CC25 (*n* = 2)	CC28 (*n* = 5)	CC221/234 (*n* = 27)	CC233/379 (*n* = 8)	Undetermined CC (*n* = 85)	
1/2	—	—	—	—	2 (25)^a^	—	2 (1.42)
2	4 (28.57)^a^	—	—	—	2 (25)^a^	—	6 (4.26)
3	—	2 (100)^a^	—	—	—	3 (3.53)	5 (3.55)
4	—	—	—	—	4 (50)^a^	3 (3.53)	7 (4.96)
5	—	—	—	—	—	4 (4.71)	4 (2.84)
7	—	—	—	—	—	4 (4.71)	4 (2.84)
8	—	—	5 (100)^a^	8 (29.63)^a^	—	1 (1.18)	14 (9.93)
9	4 (28.57)^a^	—	—	—	—	4 (4.71)	8 (5.67)
11	—	—	—	—	—	1 (1.18)	1 (0.71)
16	—	—	—	—	—	1 (1.18)	1 (0.71)
18	—	—	—	—	—	2 (2.35)	2 (1.42)
19	—	—	—	8 (29.63)^a^	—	5 (5.88)	13 (9.22)
23	—	—	—	—	—	1 (1.18)	1 (0.71)
24	—	—	—	—	—	1 (1.18)	1 (0.71)
28	—	—	—	—	—	4 (4.71)	4 (2.84)
29	—	—	—	6 (22.22)^a^	—	1 (1.18)	7 (4.96)
30	—	—	—	—	—	1 (1.18)	1 (0.71)
31	—	—	—	—	—	4 (4.71)	4 (2.84)
Non-typeable	6 (42.86)	—	—	5 (18.52)	—	45 (52.94)^a^	56 (39.72)

^a^Statistically significant positive correlation between CCs and serotypes.

**Table 3:** Distribution of serotypes, clonal complexes (CCs), and pathotypes of 141 *S. suis* isolates in 2022.

Pathotype	Clonal complex	Serotypes																		
		1/2	2	3	4	5	7	8	9	11	16	18	19	23	24	28	29	30	31	NT
Disease associated (*n* = 117)	CC1	—	4	—	—	—	—	—	4	—	—	—	—	—	—	—	—	—	—	6
	CC25	—	—	2	—	—	—	—	—	—	—	—	—	—	—	—	—	—	—	—
	CC221/234	—	—	—	—	—	—	8	—	—	—	—	8	—	—	—	6	—	—	5
	CC233/379	2	2	—	4	—	—	—	—	—	—	—	—	—	—	—	—	—	—	—
	Undetermined CC	—	—	3	2	4	4	1	4	—	1	2	5	1	1	1	1	1	4	31
Undetermined^a^ (*n* = 21)	CC28	—	—	—	—	—	—	5	—	—	—	—	—	—	—	—	—	—	—	—
	Undetermined CC	—	—	—	1	—	—	—	—	—	—	—	—	—	—	3	—	—	—	12
Non-disease-associated (*n* = 3)	Undetermined CC	—	—	—	—	—	—	—	—	1	—	—	—	—	—	—	—	—	—	2

^a^The isolates display PCR specific products of both disease-associated and non-disease-associated markers. NT, non-typeable.

**Table 4:** Distribution of serotypes, clonal complexes (CCs), and human-associated clades (HACs) of *S. suis* isolates from nasopharyngeal swabs of slaughtered pigs.

Serotype	CC1		CC25		CC28		CC221/234		CC233/379		Undetermined CC		Total
	HAC	Non-HAC	HAC	Non-HAC	HAC	Non-HAC	HAC	Non-HAC	HAC	Non-HAC	HAC	Non-HAC	
2	—	4	—	—	—	—	—	—	2	—	—	—	6
3	—	—	2	—	—	—	—	—	—	—	—	3	5
4	—	—	—	—	—	—	—	—	4	—	3	0	7
5	—	—	—	—	—	—	—	—	—	—	1	3	4
7	—	—	—	—	—	—	—	—	—	—	2	2	4
8	—	—	—	—	5	—	—	8	—	—	—	1	14
9	—	4	—	—	—	—	—	—	—	—	1	3	8
11	—	—	—	—	—	—	—	—	—	—	—	1	1
16	—	—	—	—	—	—	—	—	—	—	—	1	1
18	—	—	—	—	—	—	—	—	—	—	—	2	2
19	—	—	—	—	—	—	8	0	—	—	5	0	13
23	—	—	—	—	—	—	—	—	—	—	1	—	1
24	—	—	—	—	—	—	—	—	—	—	—	1	1
28	—	—	—	—	—	—	—	—	—	—	1	3	4
29	—	—	—	—	—	—	—	6	—	—	—	1	7
30	—	—	—	—	—	—	—	—	—	—	—	1	1
31	—	—	—	—	—	—	—	—	—	—	—	4	4
1/2	—	—	—	—	—	—	—	—	2	—	—	—	2
NT	—	6	—	—	—	—	2	3	—	—	3	42	56
Total	0	14	2	0	5	0	10	17	8	0	17	68	141

NT, non-typeable isolates.

## Data Availability

The data supporting the findings of this study are included in the article and its supplementary materials.

## Supplementary Materials

Supplementary Materials Table S1: primer sequences used for multiplex PCR serotyping. Table S2: primer sequences for detecting clonal complexes (CCs) associated with human infections. Table S3: primer sequences for pathotyping. Table S4: primer sequences for human-associated clade (HAC) marker. Table S5: pairwise correlation between human-associated clades (HACs) with clonal complexes (CCs) and/or serotypes of *S. suis* isolates. Figure S1: nucleotide sequence alignment of the *cpsK* to differentiate serotype 1/2 or 2.
