# Supplementary material for: Serotype Distribution and Pathotypic Characteristics of Streptococcus suis Isolates from Slaughtered Pigs in a High-Density Pig Farming Area in Thailand
Source: Transbound Emerg Dis. 2024 Jul 10;2024:3186518. doi: 10.1155/2024/3186518 (PMC12016684; doi:10.1155/2024/3186518)
Supplement: Supplementary Materials — Table S1: primer sequences used for multiplex PCR serotyping. Table S2: primer sequences for detecting clonal complexes (CCs) associated with human infections. Table S3: primer sequences for pathotyping. Table S4: primer sequences for human-associated clade (HAC) marker. Table S5: pairwise correlation between human-associated clades (HACs) with clonal complexes (CCs) and/or serotypes of S. suis isolates. Figure S1: nucleotide sequence alignment of the cpsK to differentiate serotype 1/2 or 2. [file 3186518.f1.docx]

**Table S1.** Primer sequences used for multiplex PCR serotyping **(**Kerdsin et al., 2014**).**

| **PCR reaction set** | **Target serotypes** | **Nucleotide sequences (5′–3′)** | **Amplicon size (bp)** |
| --- | --- | --- | --- |
| 1 | 1 or 14 | F: AATCATGGAATAAAGCGGAGTACAG  B: ACAATTGATACGTCAAAATCCTCACC | 550 |
|  | 2 or ½ | F: GATTTGTCGGGAGGGTTACTTG  B: TAAATAATATGCCACTGTAGCGTCTC | 450 |
|  | 3 | F: TGGGAGAAGGCAGAAAGTACGAGA  B: ACCCCCAGAAGAGCCGAAGGA | 1,273 |
|  | 7 | F: GATGATTTATGGCACCCGAGTAAGC  B: AGTCACAATTGCTGGTCCTGACACC | 150 |
|  | 9 | F: GGGATGATTGCTCGACAGAT  B: CCGAAGTATCTGGGCTACTG | 300 |
|  | 11 | F: TACAGTGCTTGCAGCCCTAC  B: CGACTTGTCGTGCCCTGAT | 896 |
|  | 16 | F: TGGAGGAGCATCTACAGCTCGGAAT  B: TTTGTTTGCTGGAATCTCAGGCACC | 202 |
| 2 | 4 | F: ACTTGGAGTTGTCGGAGTAGTGCT  B: ACCGCGATGGATAGGCCGAC | 783 |
|  | 5 | F: TGATGGCGGAGTTTGGGTCGC  B: CGTAACAACCGCCCCAGCCG | 166 |
|  | 8 | F: ATGGGCGTTGGCGGGAGTTT  B: TTACGGCCCCCATCACGCTG | 320 |
|  | 12 | F: TGTGGCGATAGGACAACAGG  B: ACCAAGAAGTTTCCGCCTGA | 209 |
|  | 18 | F: CGGGGCAGTCTTACTCATGG  B: ATGACAGCGAAACGGACAGA | 432 |
|  | 19 | F: AGCAGGGTTGCGTATGGCGG  B: ACAAGCACCAGCAAAGACCGCA | 1,024 |
|  | 24 | F: ACCCGGAAAAACCAGGAGTT  B: ACCAATCAATGCCAAGCGAC | 500 |
|  | 25 | F: GGAGGAGCTGCGGGCTCATA  B: TGGCCACAACCTGGATGCGTT | 1,211 |
| 3 | 6 | F: TACGGTCTCCCTTGCCTGTA  B: AACTCAGCTAGTGCTCCACG | 325 |
|  | 10 | F: TTACGAGGGGATTCTGGGGT  B: CGGGACAACAGATGGAACCT | 153 |
|  | 13 | F: CTGGTGCTGCAATTTCGCTT  B: GCAGACTAGCTGCAGTTCCA | 1,135 |
|  | 15 | F: GCAAGAAAGCTTCCGGATGGA  B: CAAGAGAGTGTGCAACCCCA | 274 |
|  | 17 | F: ACTTGGGTTGGAATGGCGAA  B: ACCACCGAAAGTCAGGTCAC | 906 |
|  | 23 | F: TGCTCAACAAACGCAGCAAA  B: TGACTGGTACATCTGCAGCC | 454 |
|  | 31 | F: GGAGTGCTCTATGCCACCTT  B: GCATTGCCCCTACAGCAAAC | 550 |
| 4 | 21 | F: GGTGGCAAGGAGAGCAAAGT  B: ACATGGTAAGCCATTGCTGGA | 325 |
|  | 27 | F: CTACGCCAATCGAAGCCAGA  B: CCAGTAAGAAGCCTGTCGCA | 506 |
|  | 28 | F: GGACTTCGGTACCTTAGCGT  B: CTCCAGCACATTCCCGTACC | 865 |
|  | 29 | F: GTGCGGGCGTTATTTTTGGT  B: AGCCTTGCAACCCATTTCCT | 435 |
|  | 30 | F: CTTTAATTGCTTGCGCCCGT  R: ATTCGGGCTACCCATTGCAG | 170 |

**Table S2**. Primer sequences for detecting clonal complexes (CCs) associated with human infections (Hatrongjit et al., 2016).

| **Target genes** | **Nucleotide sequences (5′–3′)** | **Amplicon size (bp)** |
| --- | --- | --- |
| Hypothetical protein (*hp1*) | F: TGATTACTCCTGATTCTGGAAGCGT | 408 |
|  | R: TCCTATGACTTACCATAACGACGGT |  |
| Muramidase-like protein (*mrp*) | F: TCTCCCAAACGCCACTCTGAGC | 901 |
|  | R: GGTATGTCGCTAGCCGTTGGTGC |  |
| Collagen adhesion (*col*) | F: AGAAAATGGCCTGTTCGGAATAC | 1,586 |
|  | R: ACGGAGTCGCATCTAGCACA |  |
| Peptidase (*pep*) | F: AGACCGCGGGACCATACCCA | 1,420 |
|  | R: GGCGGCAAGGGCTGCTTAGT |  |
| Sortase (*srtBCD*) | F: TGGACGGGTAGAGTATGCAC | 2,065 |
|  | R: AGGAGGAACTAGTGGATCAC |  |

**Table S3**. Primer sequences for pathotyping (Wileman et al., 2019).

| **Target genes** | **Primer name** | **Nucleotide sequences (5′–3′)** | **Marker of:** | **Amplicon size (bp)** |
| --- | --- | --- | --- | --- |
| Copper exporting  ATPase 1 | SSU0207_0735F | TTACAAGAACAGGGCAAGACAGTCGCC | Disease association | 211 |
|  | SSU0207_0945R | GCTGCTTTATAATCTGGGTGTTCGTTG |  |  |
| Type I restriction-modification  system S protein | SSU1589_0460F | CCTTTAATGCAGGGGACAAAAGTGAGCTC | Disease association | 347 |
|  | SSU1589_0806R | CCCATAATCTTACAGTTAACTTCCTTGC |  |  |
| Putative sugar ABC transporter | SSUST30534_0368F | ATCCCCTCCCAATAAAAGATTTGGATGC | Non-disease association | 892 |
|  | SSUST30534_1259R | TTTTCGAGCTCTCCATACACTGCTTCTG |  |  |
| WhiA sporulation regulator | SSU0577_0086F | CAGGTAGTTTGGGCTTAGCTTCATCAGG | *Streptococcus suis* | 722 |
|  | SSU0577_0807R | TGGATGCTGAATTCGCAACTGGGCAATC |  |  |

**Table S4**. Primer sequences for human-associated clade marker (Adapted from Dong et al., 2021)

| **Target genes** | **Primer name** | **Nucleotide sequences (5′–3′)** | **Marker of:** | **Amplicon size (bp)** |
| --- | --- | --- | --- | --- |
| Membrane protein HAC17279 | HAC17279F | TTACAAGAACAGGGCAAGACAGTCGCC | Human-associated clade | **602** |
|  | HAC17279R | GCTGCTTTATAATCTGGGTGTTCGTTG |  |  |

**Table S5.** Pairwise correlation between human-associated clades (HACs) with clonal complexes (CCs) and/or serotypes of *S. suis* isolates in 2022. Noted that only 42 out of 141 representative isolates categorized as HAC are listed.

| ***S. suis* character** | **No. of isolates, n (%)** | **Correlation**  **(*r*-value) ^a^** |
| --- | --- | --- |
| **Clonal complex** |  |  |
| CC25 (n = 2) | 2 (100) | **0.184** |
| CC28 (n = 5) | 5 (100) | **0.294** |
| CC221/234 (n = 27) | 10 (37.04) | 0.077 |
| CC233/379 (n = 8) | 8 (100) | **0.377** |
| Undetermined CC (n = 85) | 17 (20.00) | -0.264 |
| **Serotype** |  |  |
| ½ (n = 2) | 2 (100) | **0.184** |
| 2 (n = 6) | 2 (33.33) | 0.016 |
| 3 (n = 5) | 2 (40.00) | 0.043 |
| 4 (n = 7) | 7 (100) | **0.351** |
| 5 (n = 4) | 1 (25.00) | -0.018 |
| 7 (n = 4) | 2 (50.00) | 0.076 |
| 8 (n = 14) | 5 (35.71) | 0.043 |
| 9 (n = 8) | 1 (12.50) | -0.093 |
| 19 (n = 13) | 13 (100) | **0.489** |
| 23 (n = 1) | 1 (100) | 0.129 |
| 28 (n = 4) | 1 (25.00) | -0.018 |
| Non-typeable (n = 56) | 5 (8.93) | -0.370 |
| **Clonal complex - Serotype** |  |  |
| CC25 - 3 (n = 2) | 2 (100) | **0.184** |
| CC28 - 8 (n = 5) | 5 (100) | **0.294** |
| CC221/234 - 19 (n = 8) | 8 (100) | **0.377** |
| CC221/234 - non-typeable (n = 5) | 2 (40.00) | 0.043 |
| CC233/379 - 1/2 (n = 2) | 2 (100) | **0.184** |
| CC233/379 - 2 (n = 2) | 2 (100) | **0.184** |
| CC233/379 - 4 (n = 4) | 4 (100) | **0.262** |
| Undetermined CC - 4 (n = 3) | 3 (100) | **0.226** |
| Undetermined CC - 5 (n = 4) | 1 (25.00) | -0.018 |
| Undetermined CC - 7 (n = 4) | 2 (50.00) | 0.076 |
| Undetermined CC - 9 (n = 4) | 1 (25.00) | -0.018 |
| Undetermined CC - 19 (n = 5) | 5 (100) | **0.294** |
| Undetermined CC - 23 (n = 1) | 1 (100) | 0.130 |
| Undetermined CC - 28 (n = 4) | 1 (25.00) | -0.018 |
| Undetermined CC - non-typeable (n = 45) | 3 (6.67) | -0.346 |

^a^ Numbers in boldface represent statistically significant (*p*-value <0.05) correlated with HAC status.


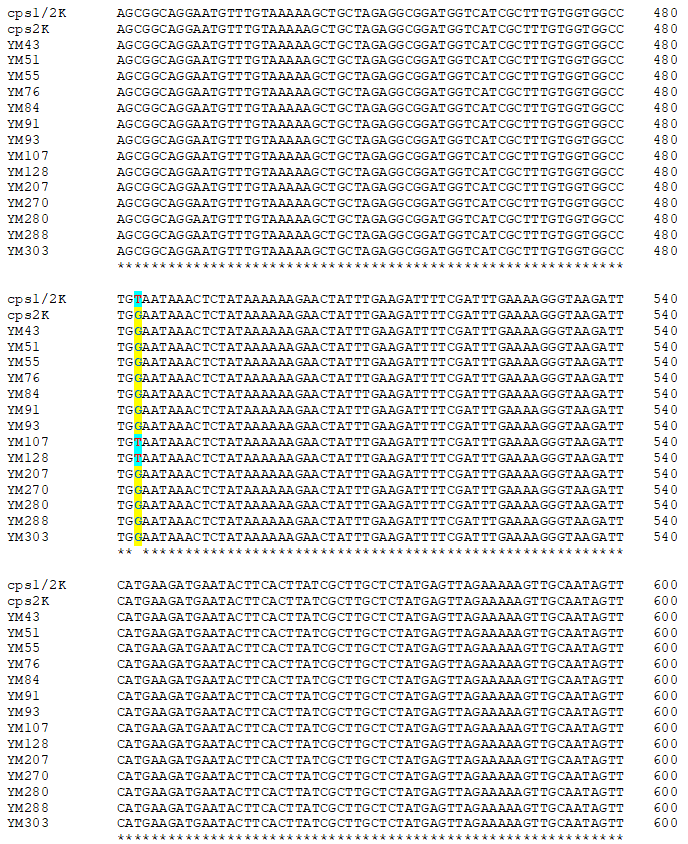


**Figure S1.** Nucleotide sequence alignment of the *cpsK* to differentiate serotype 1/2 or 2 among the 14 *S. suis* isolates. The alignment result indicated YM107 and YM128 were serotype 1/2, whereas the other 12 isolates belong to serotype 2.
